# DEHP Impairs Zebrafish Reproduction by Affecting Critical Factors in Oogenesis

**DOI:** 10.1371/journal.pone.0010201

**Published:** 2010-04-15

**Authors:** Oliana Carnevali, Luca Tosti, Claudia Speciale, Chun Peng, Yong Zhu, Francesca Maradonna

**Affiliations:** 1 Department of Marine Sciences, Polytechnic University of Marche, Ancona, Italy; 2 National Institute of Biostructures and Biosystems, Roma, Italy; 3 Department of Biology, York University, Toronto, Ontario, Canada; 4 Department of Biology, East Carolina University, Greenville, North Carolina, United States of America; Texas A&M University, United States of America

## Abstract

Public concerns on phthalates distributions in the environment have been increasing since they can cause liver cancer, structural abnormalities and reduce sperm counts in male reproductive system. However, few data are actually available on the effects of Di-(2-ethylhexyl)-phthalate (DEHP) in female reproductive system. The aim of this study was to assess the impacts of DEHP on zebrafish oogenesis and embryo production. Female *Danio rerio* were exposed to environmentally relevant doses of DEHP and a significant decrease in ovulation and embryo production was observed. The effects of DEHP on several key regulators of oocyte maturation and ovulation including bone morphogenetic protein-15 (BMP15), luteinizing hormone receptor (LHR), membrane progesterone receptors (mPRs) and cyclooxygenase (COX)-2 (ptgs2) were determined by real time PCR. The expressions of BMP15 and mPR proteins were further determined by Western analyses to strengthen molecular findings. Moreover, plasma vitellogenin (vtg) titers were assayed by an ELISA procedure to determine the estrogenic effects of DEHP and its effects on oocyte growth. A significant reduction of fecundity in fish exposed to DEHP was observed. The reduced reproductive capacity was associated with an increase in ovarian BMP15 levels. This rise, in turn, was concomitant with a significant reduction in LHR and mPRβ levels. Finally, ptgs2 expression, the final trigger of ovulation, was also decreased by DEHP. By an *in vitro* maturation assay, the inhibitory effect of DEHP on germinal vesicle breakdown was further confirmed. In conclusion, DEHP affecting signals involved in oocyte growth (vtg), maturation (BMP15, LHR, mPRs,) and ovulation (ptgs2), deeply impairs ovarian functions with serious consequences on embryo production. Since there is a significant genetic similarity between *D.rerio* and humans, the harmful effects observed at oocyte level may be relevant for further molecular studies on humans.

## Introduction

Endocrine disruptors (EDs) are able to disrupt the activity of the endocrine system and therefore modulate the metabolic activity of organs, tissues, cells and target structures [1]. Many EDs can interact with estrogen or androgen receptors and thus act as agonists or antagonists of endogenous hormones. Increasing evidence shows that EDs may also modulate the activities/expressions of steroidogenic enzymes [2]. Recent screening studies carried out in industrialized countries to detect contaminants in human urine samples have revealed the population's ubiquitous exposure to various plasticizers belonging to the group of phthalates (esters of a-phthalic acid). Di-(2-ethylhexyl)-phthalate (DEHP) is the most commonly used plasticizer in PVC formulation for a wide variety of applications including medical devices, construction products, clothing and car products. DEHP is also used in non-polymer materials such as lacquers and paints, adhesives, fillers and printing inks and cosmetics [3]. As a result, DEHP has been found everywhere in the environment, and is universally considered to be a ubiquitous environmental contaminant [4]. In the last 3 years, the number of studies reporting a relationship between exposure of environmental phthalic acid ester (PAE) and human health has rapidly increased. These studies suggest possible associations between environmental exposure to PAEs and adverse effects on human reproduction and health [5]–[7], similar to those already described for rats dosed during gestation and/or lactation with phthalates [8]. In addition, studies have shown that exposure of pregnant laboratory animals to high doses of DEHP led to the similar effects as those caused by antiandrogens [9], [10].

Follicle development, oocyte maturation and ovulation in fish are controlled by hormones, including the follicle stimulating hormone (FSH) and the luteinizing hormone (LH), as well as growth factors and hormones produced by the ovary [11]. The bone morphogenetic protein-15 (BMP15), a member of the transforming growth factor β (TGFβ) superfamily, has recently been demonstrated to prevent precocious oocyte maturation [12], [13] by inhibiting the expression of LH receptor (LHR) and membrane progestin receptors (mPRs) [14], [15] which are known to have a pivotal role in the final steps of maturation [16]. By preventing small follicles from undergoing maturation, BMP15 may be important in maintaining oocyte quality and subsequent ovulation, fertilization, and embryo development [17].

The ovulatory process, the last step of oogenesis, which ultimately leads to the rupture of the follicle wall and the release of oocytes, involves a complex series of biochemical and biophysical events. The pre-ovulatory surge of LH triggers a marked and obligatory increase in follicular prostaglandin synthesis prior to ovulation, and the cyclooxygenase (COX) enzyme is a key rate-limiting step in the biosynthesis of prostaglandins. It in fact catalyzes the conversion of arachidonic acid to prostaglandin H2, involved in the ovulation process [18].

Recently, the zebrafish and human genomes have been shown to share extensive conserved syntenic fragments and many zebrafish genes and their human homologs display structural and functional similarities. These results, in addition to providing sound elements for environmental risk assessment, can be considered a starting point for further molecular studies on humans [19], [20].

While the effects of DEHP on humans and mammalian species have largely been investigated especially in males, few data are available on its effects on the reproduction of aquatic organisms such as fish, important sentinels of environmental quality. Considering that the Environmental Protection Agency (EPA) has established a DEHP safety concentration limit in drinking water on 6 ppb (µg/l), in this study, the impact of relevant environmental concentrations [21], ranging from 0.02 to 40 µg/l, on zebrafish oocytes maturation, ovulation and fecundity, was analyzed. The molecular mechanisms of the adverse effects of DEHP were also investigated.

## Methods

### Experimental design

Adult *Danio rerio* (zebrafish) females were purchased from a commercial dealer (Acquario di Bologna, BO, IT). They were kept in aquaria at 28°C and oxygenated water. Fish were fed twice daily with commercial food (Vipagran, Sera, Germany) and other two times with *Artemia salina*. Eggs laid by parental fish were kept and grown. Six months old adult zebrafish were used for toxicological studies.

Females were exposed for three weeks, in semi-static conditions, to nominal 0.02, 0.2, 2, 20 and 40 µg/l concentrations of DEHP. In order to evaluate DEHP estrogenic potency, one group was exposed to the positive control, 17α-Ethynylestradiol (EE2 25 ng/l). To investigate potential effect of the solvent, the vehicle control (EtOH), was used as control for all experimental groups. For each concentration, the treatment was performed in three different tanks (30 fish each), at a mean density of 1 fish/l, at a constant day/night photoperiod (12L/12D).

### Chemicals

A technical grade DEHP (purity 99%) used in all experiments was obtained from Supelco (Bellafonte, PA, USA). Stock solution of 1 mg/ml was prepared dissolving both DEHP and 17α-Ethynylestradiol (EE2, purity 98% Sigma) in ethanol.

### Reproductive performance

At the end of the three weeks' exposure, females (n = 10) from each experimental group were transferred to spawning tanks containing non contaminated water together with non treated zebrafish males (ratio: 10female/7males). Fecundity, defined as daily number of fertilized eggs (embryos), was determined for the next 14 days. For each pollutant concentration, three spawning tanks were set up.

### Percent of follicles of each stage of development

Following exposure, adult females (n = 5) from each experimental group were sacrificed and oocyte follicles stages determination was performed. The ovary of zebrafish is in fact asynchronous and oocytes at different stages of development are simultaneously present [22]. The oocytes were divided into three different groups according to their sizes: previtellogenic (0.15–0.34 mm Ø), vitellogenic (0.35–0.69 mm Ø) and postvitellogenic (0.70–0.75 mm Ø). Follicles were manually isolated using micro tweezers under a microscope equipped with a micrometric scale in the objective. Each follicle stage was expressed as a percent of the total number of follicles from both ovaries of each female used.

### Enzyme-linked immuno sorbent assay (ELISA)

Rabbit anti-zebrafish vtg polyclonal antibody was purchased by Biosense Laboratories AS (Thormøhlensgt. 55, N-5008 Bergen, Norway). The assay to determine vtg concentration was performed in the plasma of 5 fish. Standard curves were obtained by adding increasing doses of vtg from 10 to 1280 ng [23]. A reliable calibration curve enables the antigen titer (vtg) to be measured in all culture media.

### Gene expression

Total RNA was extracted from 5 ovaries using TRI REAGENT TM (Sigma) following manufacturer's instructions. The cDNA synthesis and real time PCR assay were performed using the SYBR green described as previously [24]. Quantifications of LHR, mPRα, mPRβ, ptgs2 gene expression were normalized using ARP (Acidic Ribosomal Protein, a house-keeping gene) in each sample in order to standardize the results by eliminating variations in mRNA and cDNA quantity and quality. In order to amplify, LHR, mPRα, mPRβ, ptgs2 and ARP gene, the following primer pairs were used: FLHR 5′ GGCGAAGGCTAGATGGCACAT3′ RLHR 5′TCGCCATCTGGTTCATCATA3′; FmPRα 5′ CGGTTGTGATGGAGCAGATT3′; RmPRα 5′ AGTAGCGCCAGTTCTGGTCA 3′; FmPRβ 5′ ACA ACGAGCTGCTGAATGTG3′, RmPRβ 5′ATGGGCCAGTTCAGAGTGAG 3′, Fptgs2 5′TGGATCTTTCCTGGGTGAAGG3′; Rptgs2 5′GAAGCTCAGGGGTAGTGCAG3′; FARP 5′TTCCTCGGTATGGAGTCCT3′, RARP 5′-TGGGGCAATGATCTTGATCTT3′.

### Western blot analysis

Ovary homogenates were prepared, electrophoresed, and transferred to PVDF membrane. The membranes were then probed with anti-BMP15, anti-mPRα and anti-mPRβ antisera as previously reported [25]. Data were normalized against β tubulin protein levels. Anti-β-tubulin antibody (1 g/ml) (Gene Tex, Inc.) was used to normalize the sample loading. The antibody reaction was visualized with chemiluminescent reagent for Western blot. The densitometric analysis was performed by ImageJ software for Windows.

### Follicles *in vitro* maturation

Maturation assays were performed as previously described [13]. Briefly, gravid female zebrafish were anesthetized using 3-aminobenzoic acid ethyl ester (Sigma-Aldrich Canada Inc., Oakville, ON, Canada) and decapitated. The ovaries were removed; follicles were staged according to their size and stage IIIB oocyte were collected. These oocytes were then pre-incubated for 4 hrs with 10 nM or 100 nM DEHP before the addition of 17,20β dihydroxy-4-pregnen-3one (17,20βP) (100 ng/ml, Sigma Aldrich, Milan Italy). Additional groups were incubated with DEHP and 17,20βP simultaneously. The rate of maturation indicated by the germinal vesicle breakdown (GVBD) was scored after 12 hrs post incubation. Each treatment was conducted with approximately 20 follicles per well and all experiments were carried out at least three times.

### Data Analysis

Statistical analysis was performed with GraphPad Prism version 5.00 for Windows, GraphPad Software, San Diego California USA. Normal distribution of any variable analysed was checked by Kolgomorov–Smirnov test. Data for dose-response studies were analyzed for statistical significance by one-way ANOVA. Bonferroni's multiple comparison tests were used to determine differences among groups. Significance was set at p<0.05.

## Results

### Effects of DEHP exposure in female zebrafish on oocyte growth and maturation

**Figure 1:**
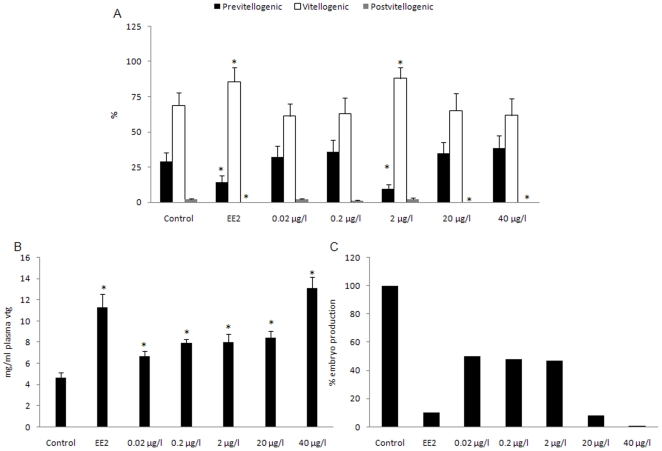
Effects of DEHP exposure in female zebrafish on oocyte growth and embryo production. A, Percentages of pre-vitellogenic, vitellogenic, post-vitellogenic oocytes in female (n = 5) exposed to DEHP (0.02, 0.2, 2, 20 and 40 µg/l) or EE2 (25 ng/l). Asterisks* indicate statistical significant differences set at P<0.05. Data were shown as mean ± SEM. B, Increase of plasma vitellogenin levels in female zebrafish (n = 3) exposed to DEHP (0.02, 0.2, 2, 20 and 40 µg/l) or EE2 (25 ng/l). Data were shown as mean ±SD. Asterisks* indicate statistical significant differences compare to control group (p<0.05). C, Reduction of total embryos in female zebrafish exposed to (0.02, 0.2, 2, 20 and 40 µg/l) DEHP or EE2 (25 ng/l).

Treatment with EE2 or the 2 µg/l DEHP dose led to a significant increase in the number of vitellogenic oocytes. This increase was associated with a significant decrease in pre-vitellogenic oocytes observed in the same experimental groups; EE2 and 2 µg/l DEHP shifted pre-vitellogenic oocytes towards vitellogenic induction. Interestingly, no post-vitellogenic oocytes were found in the EE2, 20 or 40 µg/l DEHP exposed females, (Figure 1A). In all treated groups, the GSI (gonad-somatic index) became higher, although not significantly except for the EE2 group where a significant increase was found (data not shown). A significant increase in vtg levels in the plasma of treated females was observed with the highest induction found with 40 µg/l DEHP, clearly showing the estrogenic activity of DEHP (Figure 1B).

After three weeks DEHP exposure, females were paired with control males and in the following 14 days, the embryos were collected and counted daily. The fecundity was remarkably compromised by DEHP at all doses tested. The most dramatic effects were observed at the highest dose of DEHP where the number of embryos was about 1% of the embryos produced by the control (Figure 1C). The number of embryos collected in females exposed to EE2 was similar to that obtained with 20 µg/l DEHP.

### Effects of DEHP on *in vitro* GVBD

**Figure 2:**
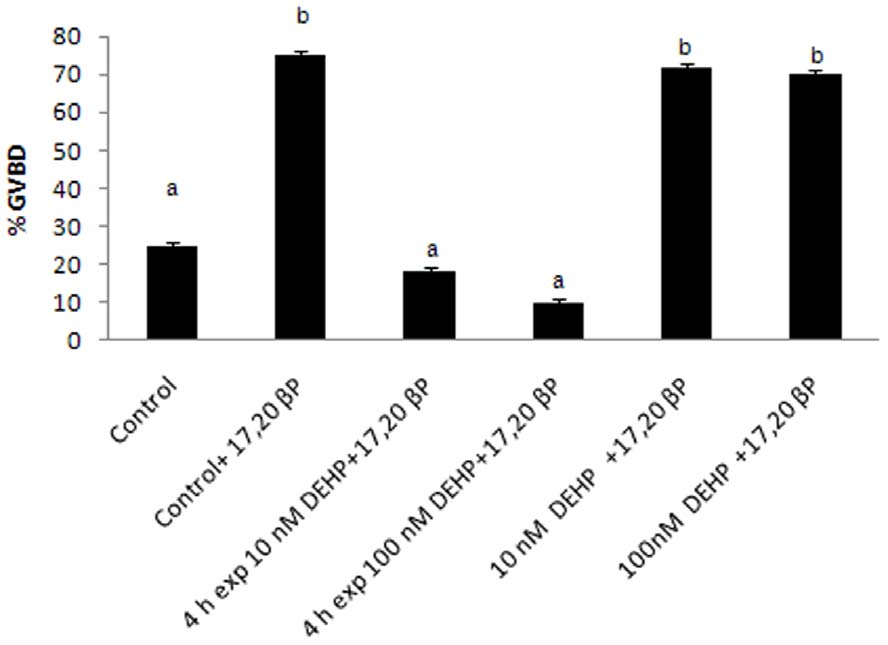
Pre-exposure of DEHP inhibits steroid-induced germinal vesicle breakdown (GVBD) *in vitro*. Stage IIIB follicles were collected from female zebrafish and were pre-exposed to DEHP for 4 hrs before addition of 17,20 βP, or simultaneously exposed to DEHP and 17,20 βP. Data represent mean ± SD of three replicate wells from one experiment. The experiment was repeated three times with similar results. Different letters indicate statistical significant differences compare to control group (p<0.05).

To further confirm the effect of DEHP on oocyte maturation, *in vitro* maturation assays were performed. Approximately 75% stage IIIB follicles underwent GVBD following treatment with 17,20βP alone for 12 hrs. In contrast, the steroid-induced oocyte maturation (GVBD) was inhibited significantly when stage IIIB oocytes were pre-exposed with two different doses of DEHP for 4 hrs prior to the treatment of 17,20βP, compared to 17,20βP treatment alone. When DEHP and 17,20βP were added simultaneously, no inhibition of steroid-induced GVBD was observed (Figure 2).

### Effects of DEHP exposure on ovarian BMP15 protein level

Since exposure to DEHP caused a strong inhibition in oocyte maturation, we tested the effect of DEHP on BMP15 known to be involved in this processes.

**Figure 3:**
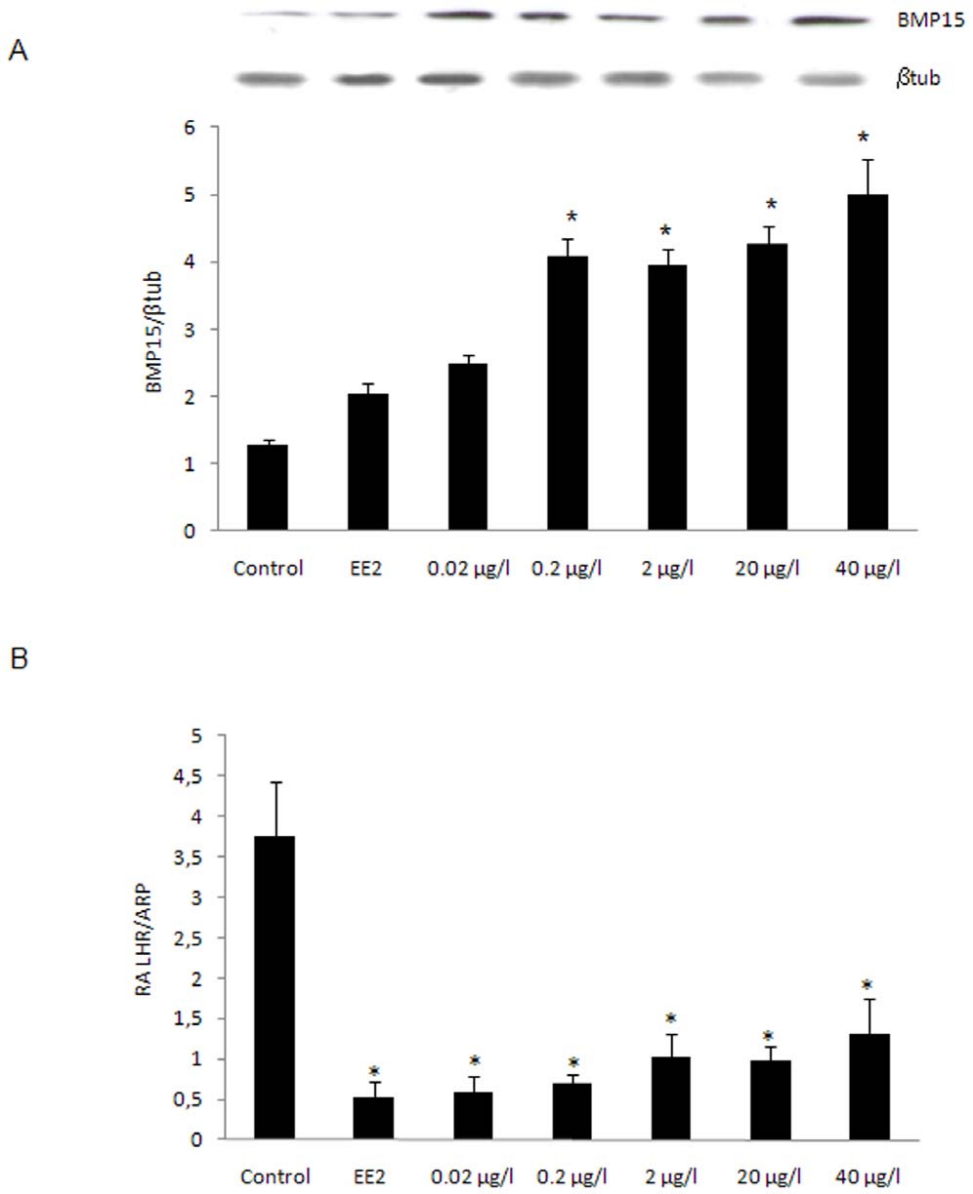
Up-regulation of ovarian BMP15 protein level and down-regulation of LHR gene expression in female zebrafish exposed to DEHP. A, BMP15 protein levels in vehicle control (EtOH), DEHP (0.02, 0.2, 2, 20 and 40 µg/l) or EE2 (25 ng/l) treated fish. Insert shows a representative Western blot while the graph represents densitometric analysis of BMP15 normalized using β-tubulin. Data were expressed as the mean±SD from at least four separate experiments. Asterisks* denote exposure groups that are significantly different from the control group (p<0.05). B, Down regulation of ovarian LHR gene expression normalized using ARP gene in females exposed to DEHP (0.02, 0.2, 2, 20 and 40 µg/l) or EE2 (25 ng/l.) Data were expressed as mean±SD from at least four independent experiments. Asterisks* indicate statistical significant differences respect to control group (p<0.05).

A significant dose-dependent increase in BMP15 protein was observed in all groups treated with DEHP except for the 0.02 µg/l dose and EE2 groups (Figure 3A).

### Effects of DEHP exposure on ovarian LHR gene expression

A significant decrease in LHR mRNA levels was observed in the oocytes of females exposed to DEHP or EE2 compared to the vehicle control group (p<0.05). The lowest expression of LHR was obtained in fish exposed to EE2, or DEHP being the lowest doses (0.02 and 0.2 µg/l) the most injurious (Figure 3B).

### Effects of DEHP on ovarian mPRs gene and protein expression

**Figure 4:**
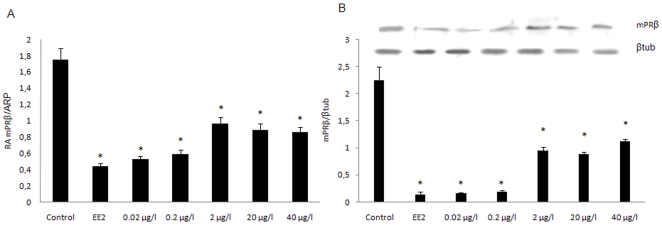
Effects of DEHP exposure on the expressions of ovarian mPRβ in female zebrafish. A, Female zebrafish were exposed to DEHP (0.02, 0.2, 2, 20 and 40 µg/l) or EE2 (25 ng/l). Data were shown as mean ± SD. Asterisks* indicate statistical significant differences to control group (p<0.05). B, Insert shows a representative Western blot while the graph represents densitometric analysis of mPRβ normalized using β-tubulin. Data are expressed as the mean ±SD from at least four separate experiments. Asterisks* denote exposure groups that are significantly different from the control group (p<0.05).

A significant decrease in both mPRβ mRNA (Figure 4A) and protein (Figure 4B) levels was observed in oocytes of females exposed to DEHP at all doses or EE2 compared to those in the vehicle control group. The lowest expression of mPR was obtained in fish exposed to EE2, or DEHP at doses of 0.02 and 0.2 µg/l. No significant variations in mPRα gene and protein expression was observed using the same treatments (data not shown).

### Effects of DEHP exposure on ovarian cyclooxygenase-2 gene expression

**Figure 5:**
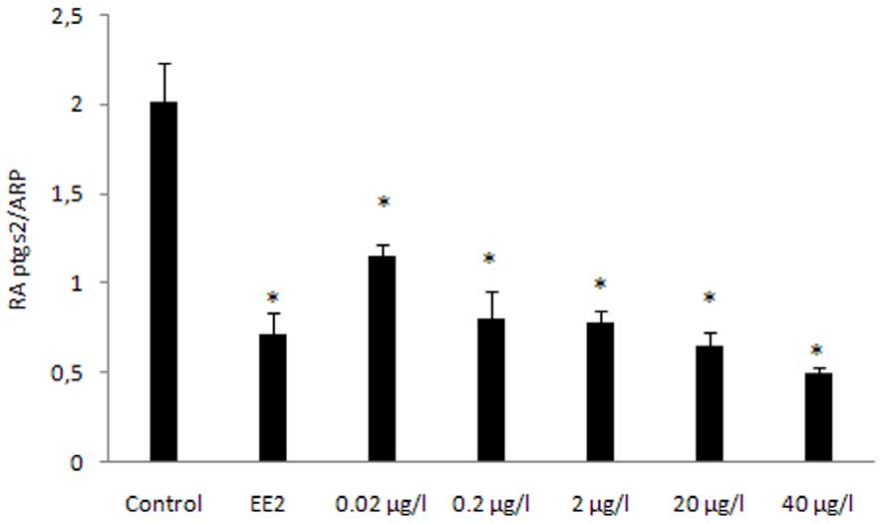
Inhibition of DEHP on ovarian ptgs2 gene expression. Female zebrafish were exposed to EE2 (25 ng/l), or DEHP at doses of 0.02, 0.2, 2, 20 or 40 µg/l. The expression of ptgs2 gene was normalized using ARP gene. Data were shown as mean±SD. Asterisks* indicate statistical significant differences compare to control group (p<0.05).

Compared to controls, a significant dose-dependent decrease in the expression of cyclooxygenase 2 (ptgs2) gene was observed with lowest levels at dose of 40 µg/l (Figure 5).

## Discussion

In this study, we provide evidence that environmentally relevant concentrations [21] of DEHP interfere with zebrafish reproductive performance, representing a concrete risk for the aquatic population living in polluted areas.

We found that exposure of female zebrafish to DEHP or EE2 led to a significant increase of circulating levels of vitellogenin. Since estrogen is well known for its effect on promoting vitellogenesis, this observation supports our hypothesis that DEHP has estrogenic effects in zebrafish. However, in fish exposed to the highest dose DEHP, there was a significant decrease in the number of post-vitellogenic oocytes and the number of ovulated eggs was dramatically decreased by all treatments.

According to a model proposed by Nagahama and collaborators [26], vitellogenesis and oocyte maturation are regulated primarily by FSH and LH, respectively. During oocyte maturation, LH induces 17,20βP production [27] and enhances the expression of membrane progestin receptors [28], [29]. Studies in zebrafish have demonstrated that the effects of these hormones may be modulated and/or mediated by locally produced regulators, such as TGFβ family members [12]. Since a member of this family, BMP15, plays a physiological role in the zebrafish ovary by preventing precocious oocyte maturation [13], in the present study, its levels were determined in the gonad of DEHP treated females and the protein level was seen to significantly increase, representing one of the possible factors responsible for the observed block in oocyte maturation. In contrast to the increase in BMP15 level, we found that DEHP caused an inhibition of the LHR and mPRβ expression. The worst effect on these maturation factors was induced by the lowest doses of DEHP, behaving as EE2.

It has been reported that BMP15 inhibited expression of mPRβ, but not mPRα expression [14], [25] and might also suppress LHR expression [17]. The mPRβ has been demonstrated to be involved in initializing the resumption of meiosis during *Xenopus* oocyte maturation [30] and in regulating *in vitro* maturation of pig cumulus-oocyte complexes [31]. Moreover, while previous studies have shown that microinjection of mPRα antisense nucleotide in oocytes resulted in partial inhibition of 17,20βP-induced oocyte maturation in goldfish [28], similar experiments conducted on zebrafish oocytes using the mPRβ subtype resulted in complete inhibition of oocyte maturation, suggesting that mPRβ has a key role in the control of this process in zebrafish [29]. The lack of egg production observed in the present study, may be due to reduction of mPRβ subtype by DEHP exposure. In addition, the differences in expression between the two mPRs here observed, might also be due to the lower frequency of stage IV follicles in treated females where the α isoform is commonly more abundant [32], [33].

The *in vivo* results were supported by the *in vitro* GVBD inhibition by DEHP which occurred only when the oocytes were exposed to this contaminant four hours before the incubation with 17,20βP, while no effects were observed when DEHP and 17,20βP were concomitantly added to oocyte cultures. These results suggest that DEHP may act on the synthesis of local factors involved in GVBD contrasting oocyte maturation when 17,20βP was added a few hours later.

About ovulation, this process is controlled by prostaglandins which are synthesized under the influence of 17,20βP. In this regard, a relationship was also demonstrated between ovarian ptgs2, the gene coding for the enzyme essential for the ovulation process [34], and the lack of ovulation in fish exposed to DEHP, being the highest doses the most detrimental.

Similar effects on vitellogenesis and ovulation, as well as on the expressions of LHR, mPRβ and ptgs2 caused by DEHP or EE2, suggest that these two chemicals work in a similar way inhibiting the maturation/ovulation process, as already found for EE2 in mammals [35]. The inhibition of ptgs2 gene transcription may lead to a reduction in the cyclooxygenase (COX) products, the prostaglandins, which in turn are essential for vertebrate ovulation [34], [36]. On the contrary, BMP15 expression was induced by all doses of DEHP, except for the lowest dose, but not by EE2. Therefore, it is possible that DEHP may have additional functions which are not related to its estrogenic activity. It remains to be determined how DEHP regulates the expression of BMP15.

In summary, the data presented here provide new insight into the molecular control of oogenesis by phthalates in zebrafish. We can conclude that all environmental relevant doses of DEHP affect vitellogenesis, demonstrating its estrogenic potency. Different dose-related effects have been observed in relation to maturation and ovulation process. The lowest doses have a stronger negative effects on signals inducing maturation (LHR and mPRβ), while the highest doses have a greater impact on the inhibition of ovulation (ptgs2). The results of this study, both *in vivo* and *in vitro*, clearly demonstrate that all doses of DEHP strongly impair oocyte maturation and ovulation by influencing the expression of factors involved in these processes. These results could help to build a vertebrate integrated model on the effects of this environmentally relevant compound during oogenesis, an emerging field of investigation.
